# Comparison of diagnostic performance between dynamic versus static adenosine-stress myocardial CT perfusion to detect hemodynamically significant coronary artery stenosis: A prospective multicenter study

**DOI:** 10.1097/MD.0000000000030477

**Published:** 2022-09-09

**Authors:** Ji Won Lee, Yeon Hyeon Choe, Sung Mok Kim, Jin-Ho Choi, Seongyong Pak, Ki Seok Choo, Jeong Su Kim, Chong Eun Lee, Yun-Hyeon Kim

**Affiliations:** a Department of Radiology, Pusan National University School of Medicine and Medical Research Institute, Pusan National University Hospital, Busan, Korea; b Department of Radiology, Sungkyunkwan University School of Medicine, Seoul, Korea; c Emergency Medicine and Cardiovascular Imaging Center, Samsung Medical Center, Sungkyunkwan University School of Medicine, Seoul, Korea; d Department of Biomedical Engineering, Asan Medical Institute of Convergence Science and Technology, Asan Medical Center, University of Ulsan College of Medicine, Seoul, Korea; e Department of Radiology, Pusan National University School of Medicine and Medical Research Institute, Pusan National University Yangsan Hospital, Yangsan-si, Gyeongsangnam-do, Korea; f Department of Internal Medicine, Pusan National University School of Medicine and Medical Research Institute, Pusan National University Yangsan Hospital, Yangsan-si, Gyeongsangnam-do, Korea; g Department of Radiology, Chonnam National University Hospital, Chonnam National University Medical School, Gwangju, Korea.

**Keywords:** coronary stenosis, multidetector computed tomography, myocardium, perfusion

## Abstract

Myocardial computed tomography perfusion (CTP) imaging is a noninvasive method for detecting myocardial ischemia. This study aimed to compare the diagnostic performance of dynamic and static adenosine-stress CTPs for detecting hemodynamically significant coronary stenosis. We prospectively enrolled 42 patients (mean age, 59.7 ± 8.8 years; 31 males) with ≥40% coronary artery stenosis. All patients underwent dynamic CTP for adenosine stress. The static CTP was simulated by choosing the seventh dynamic dataset after the initiation of the contrast injection. Diagnostic performance was compared with invasive fractional flow reserve (FFR) <0.8 as the reference. Of the 125 coronary vessels in 42 patients, 20 (16.0%) in 16 (38.1%) patients were categorized as hemodynamically significant. Dynamic and static CTP yielded similar diagnostic accuracy (90.4% vs 88.8% using visual analysis, *P* = .558; 77.6% vs 80.8% using quantitative analysis, *P* = .534; 78.4% vs 82.4% using combined visual and quantitative analyses, *P* = .426). The diagnostic accuracy of combined coronary computed tomography angiography (CCTA) and dynamic CTP (89.6% using visual analysis, *P* = .011; 88.8% using quantitative analysis, *P* = .018; 89.6% using combined visual and quantitative analyses, *P* = .011) and that of combined CCTA and static CTP (88.8% using visual analysis, *P* = .018; 90.4% using quantitative analysis, *P* = .006; 91.2% using combined visual and quantitative analyses, *P* = .003) were significantly higher than that of CCTA alone (77.6%). Dynamic CTP and static CTP showed similar diagnostic performance in the detection of hemodynamically significant stenosis.

## 1. Introduction

Coronary computed tomography angiography (CCTA) is one of the diagnostic methods for the assessment of coronary artery disease (CAD) with high diagnostic accuracy.^[1,2]^ However, the hemodynamic significance of the stenosis cannot be determined with CCTA, especially the hemodynamic significance of indeterminate coronary stenosis.^[3–5]^ Therefore, in patients with stable CAD or chest pain, the interventional cardiologist encounters coronary stenosis of 40% to 90% and a decision has to be made with regards to revascularization.^[6]^ In this process, functional evaluation is necessary when considering the physiological evaluation of coronary artery stenosis to improve decision-making and clinical outcomes for patients undergoing coronary revascularization.^[7–9]^

Myocardial computed tomography perfusion (CTP) imaging is an emerging noninvasive method for detecting myocardial ischemia with reasonable accuracy.^[10,11]^ Using dynamic CTP, both visual and quantitative analyses (e.g., myocardial blood flow [MBF] and myocardial blood volume) of the myocardium is possible. However, a high radiation dose is a major disadvantage for dynamic CTP compared to static CTP, which is a single-phase data acquisition during CTP.^[11]^ In contrast, static CT is limited to a single dataset and does not provide a comprehensive blood flow analysis.^[12]^

A previous study demonstrated that the diagnostic accuracy of semiquantitative parameters such as peak enhancement of single-shot CTP was comparable to the diagnostic accuracy of quantitative parameters including MBF and upslope using dynamic CTP.^[13]^ However, the study included a considerable number of patients with nonsignificant stenosis, and the diagnostic accuracy of visual perfusion defect assessment between dynamic CTP and single-shot CTP was not estimated. Although several studies have demonstrated the diagnostic accuracy of dynamic CTP or static CTP, there have been no reports comparing the diagnostic accuracy of both visual assessment and quantitative parameters between dynamic and static CTPs.

Therefore, the present study aimed to compare the diagnostic performance of both visual and quantitative analyses between dynamic and static CTPs to detect hemodynamically significant coronary artery stenosis and to evaluate the diagnostic capabilities of these parameters in comparison with those of CCTA in patients with ≥40% coronary artery stenosis.

## 2. Methods

### 2.1. Study population

**Figure 1.**
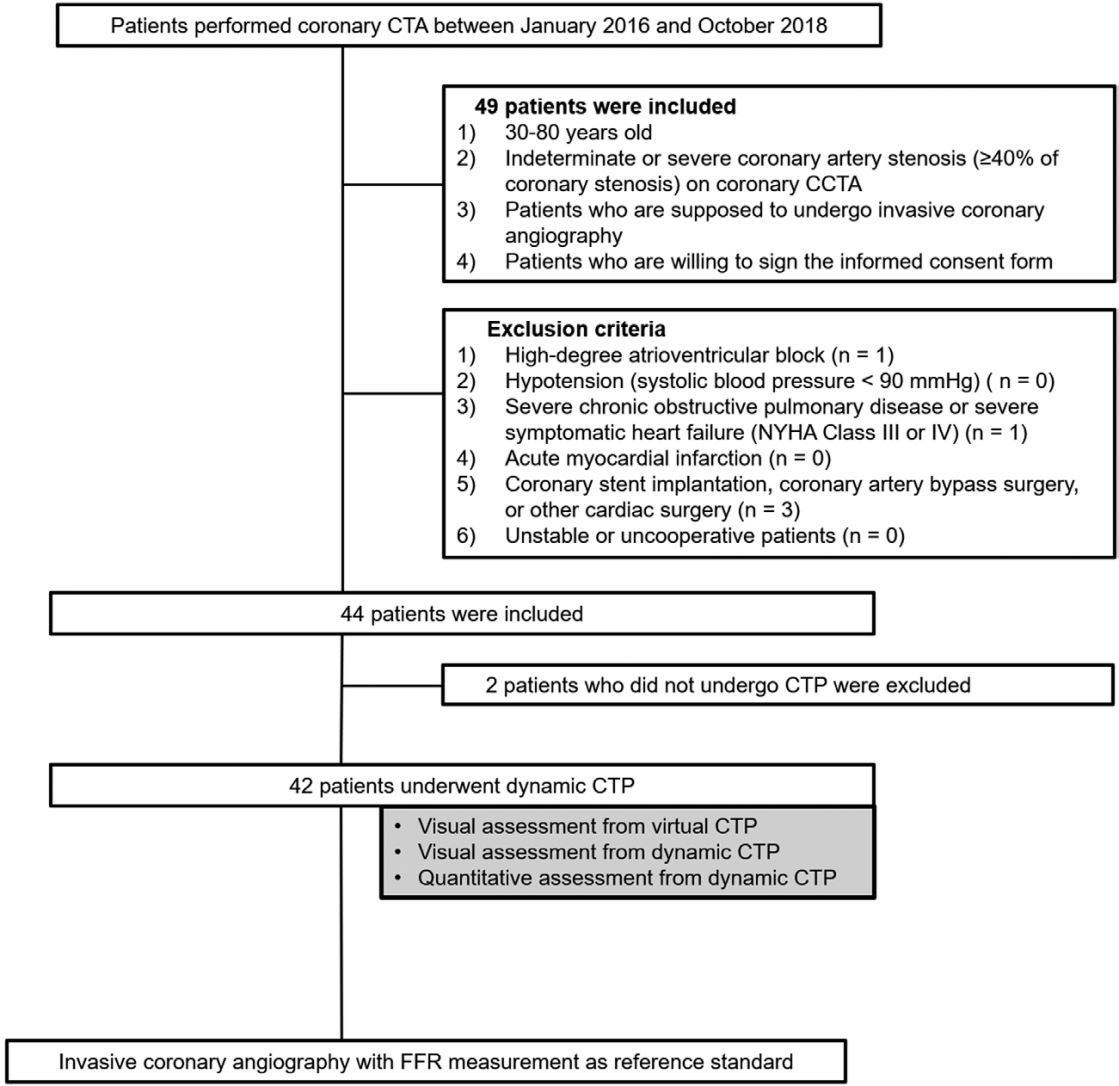
Patient selection. CCTA = coronary computed tomography angiography, CTP = computed tomography perfusion, FFR = fractional flow reserve, NYHA = New York Heart Association.

This prospective multicenter study was approved by the institutional review board of each participating institution and informed consent was obtained from all participants. Patients from 3 tertiary hospitals in XXX (XXX Hospital; XXX Hospital; XXX Hospital) who underwent CCTA between January 2016 and October 2018 were included in the study. The inclusion criteria were patients aged 30 to 80 years; with ≥40% coronary artery stenosis detected using CCTA; who were supposed to invasive coronary angiography; and who were willing to sign the informed consent form. Exclusion criteria were patients with high-degree atrioventricular block; hypotension (systolic blood pressure <90 mm Hg); severe chronic obstructive pulmonary disease or severe symptomatic heart failure (New York Heart Association Class III or IV); acute myocardial infarction; history of myocardial infarction, coronary stent implantation, coronary artery bypass surgery, or other cardiac surgery; and unstable or uncooperative patients. Out of the 44 patients enrolled in this study, 2 patients who did not undergo CTP were excluded. Finally, 42 patients (mean age, 59.7 ± 8.8 years; 31 males) were included in the study (Fig. 1).

### 2.2. Image acquisition and reconstruction

#### 2.2.1. CCTA.

Patients underwent CCTA using a second-generation (n = 37, SOMATOM Definition Flash; Siemens Healthineers, Forchheim, Germany) and a third-generation (n = 5, SOMATOM FORCE, Siemens Healthineers) dual-source CT scanner. Specific CCTA protocol is provided in the online supplementary material, Supplemental Digital Content 1, http://links.lww.com/MD/H249.

Image data were reconstructed using 0.6-mm slices and transferred to a workstation (Syngo VIA; Siemens Healthineers).

#### 2.2.2. CTP scan.

**Figure 2.**
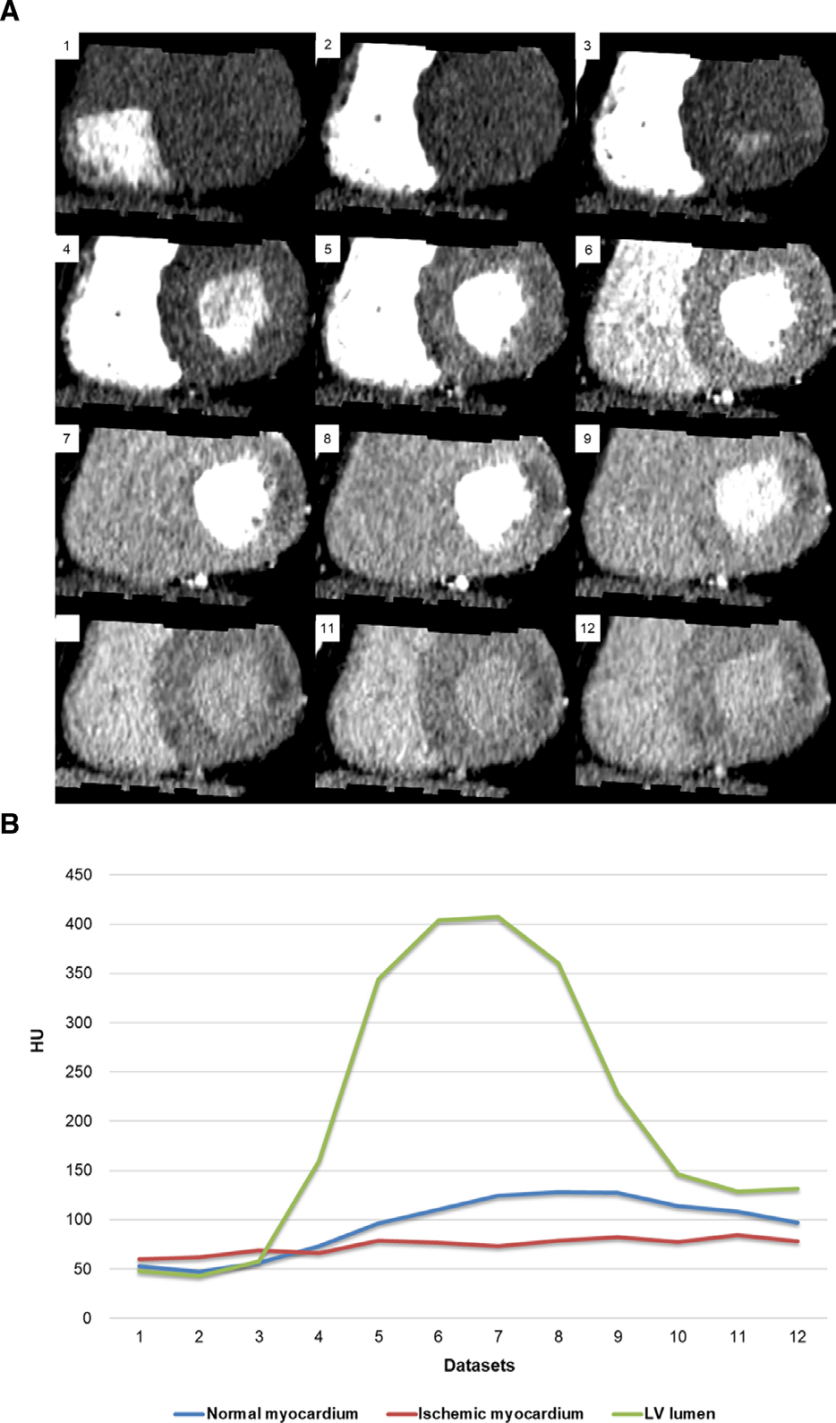
A representative image set selection for virtual static CTP from dynamic image sets. Dynamic CTP performed with 12 phases during 30 seconds in a 49-year-old man with severe stenosis of the left circumferential coronary artery. (A) Dynamic CTP images showing transmural perfusion defect (arrow) in the inferior lateral wall of the mid-left ventricle. (B) Time-attenuation curve demonstrating attenuation numbers of the normal myocardium, ischemic myocardium, and LV lumen. The maximal difference between the normal and ischemic myocardium was seen in the seventh dynamic image set (16.4 s). CTP = computed tomography perfusion, HU = hounsfield unit, LV = left ventricle.

A second-generation dual-source CT scanner was used for CTP at all centers. Adenosine (0.14 mg/kg/min) was infused into the antecubital vein for 3 minutes for all patients. After 2 minutes 54 seconds from the start of adenosine infusion, 50 mL of iohexol 350 mg I/mL (XXX 350) was administered into the antecubital vein in the opposite arm at a rate of 5 mL/s, followed by 30 mL of saline flush. A dynamic CTP scan was initiated 3 minutes after the start of the adenosine infusion. It was performed in a shuttle mode during the systole at 200 ms after the R peak with an acquisition time of 30 seconds. Given a detector width of 38 mm and a 10% overlap between both acquisition ranges, the anatomic coverage of this CTP scan was 73 mm. The specific parameters were as follows: tube voltage, 100 kVp; tube current-time product, 330 mA/rotation; gantry rotation time, 285 ms; and detector collimation, 2 × 64 × 0.6 mm. CT data were reconstructed at 1.5-mm slice thickness using a smooth kernel (B23f), and all reconstructed images were anonymized and transferred to a workstation (Syngo VIA; Siemens Healthineers). Images with a slice thickness of 3 mm and an increment of 1.5 mm were displayed and used for image analysis using this workstation. To determine the best dynamic image set for static CTP, we obtained the time-attenuation curve from 9 patients with functionally significant coronary stenosis with visually accessible perfusion defect and without 3 vessel disease. Next, we calculated the maximum attenuation difference between normal and ischemic myocardium during 30 seconds of dynamic scanning (Fig. 2) (Table S1, Supplemental Digital Content 1, http://links.lww.com/MD/H249). Our results revealed that static CTP images were determined as the seventh dynamic dataset of 16.1 to 21.0 s after the initiation of contrast injection among the 10 to 14 dynamic CTP datasets.

### 2.3. Image analysis

Two cardiac radiologists (XXX and XXX) trained in cardiac CT for 10 years and 19 years, respectively, blinded to the results of invasive coronary angiography, independently performed coronary artery stenosis analysis on CCTA and visual analysis of CTP images. Disagreements were resolved by consensus for visual assessments. Quantitative analysis of CTP images was performed by a cardiac radiologist (XXX), blinded to the results of invasive coronary angiography, CCTA. Dynamic and static CTP images were anonymized and they were reviewed with a one-month interval. A total of 125 vessel territories were analyzed, except for one right coronary artery territory that was partially scanned on the CTP in 1 patient. The excluded territory had no coronary artery stenosis on the CCTA.

#### 2.3.1. CCTA analysis.

First, the image quality of each coronary artery was graded per vessel level using a 4-point scale as follows: excellent (no motion artifacts); good (minor blurring artifacts); fair (moderate blurring artifacts); and poor (significant blurring or doubled appearance of the structure).^[14]^ Grade 4 was considered nonevaluable image quality.

Subsequently, coronary artery stenosis was assessed at the vessel level. Coronary artery stenosis was graded as no, minimal, mild, moderate, severe stenosis, and total occlusion, corresponding to stenosis of 0, ≤25%, 26% to 50%, 51% to 75%, 76% to 99%, and 100%, respectively, as recommended by the Society of Cardiovascular Computed Tomography.^[15]^ Discrepancies between the 2 observers were settled by consensus.

#### 2.3.2. CTP analysis.

A narrow window (200 Hounsfield units [HU]) and level (100 HU) settings were used for the CTP analysis. However, adjustments of the window-level settings were performed at the discretion of the observer, as deemed necessary for the best visualization. When 50% or more of a segment was out of the scan range, the segment was excluded due to incomplete coverage. The image quality of static and dynamic CTPs was assessed per vessel level using a 4-point scale as follows: excellent (no artifacts); good (no or minor artifacts); moderate (moderate artifact); poor (severe artifact). Grade 4 was considered a nonevaluable image quality.^[16]^

For visual assessment of static CTP images, hypo-attenuated areas were defined as perfusion defects if their locations could be classified according to the coronary artery territories. The presence or absence of perfusion defects was recorded according to coronary artery territories based on the 16-segment model, excluding the apical segment.^[17]^ For quantitative assessment, regions of interest were manually drawn on the short-axis images of the static CTP. Myocardial attenuation was measured for each segment and the lowest value was chosen to represent perfusion for each vascular territory.

For visual assessment of dynamic CTP images, perfusion defects were detected using the color map of the MBF and dynamic image datasets. Among abnormal lesions on the color map, a perfusion defect was defined as a hypo-attenuated area that lasted for more than 6 heartbeats, and its location was classified according to coronary artery territories on dynamic CTP datasets. MBF was calculated based on the maximum slope from dynamic datasets at each segmental level using dedicated software for myocardial perfusion analysis (Syngo VIA). The lowest value among the vascular territories was used.

For combined visual and quantitative analysis, the presence of a perfusion defect was determined when there was a perfusion defect in one or more of these 2 analyses.

### 2.4. Radiation dose

The volume CT dose index and dose-length product (DLP) were obtained from the automatically generated patient protocol. The effective radiation dose of CTP was calculated as the DLP multiplied by the cardiac-specific conversion factor (k = 0.014 mSv · mGy^-1^cm^-1^).^[18]^

### 2.5. Invasive coronary angiography

Invasive coronary angiography and CTP were performed at an interval of 30 days (7.4 ± 8.7 days; range, 0–30 days). Invasive coronary angiography using the standard techniques was performed by experienced interventional cardiologists at each center visually identified coronary stenosis on multiple projections in a blinded fashion. Fractional flow reserve (FFR) was measured for coronary artery stenosis with ≥40% luminal narrowing during intravenous adenosine infusion (0.14 mg/kg/min for 2 minutes). Lesions with FFR ≤0.8, or >90% luminal stenosis on invasive coronary angiography were classified as functionally significant.^[19,20]^

### 2.6. Statistical analysis

Statistical analyses were performed using PASW Statistics (ver. 18.0; SPSS Inc., Chicago, IL, USA) and MedCalc (ver 19.2.1; MedCalc Software Ltd, Ostend, Belgium). Categorical variables, presented as percentages, were compared using the chi-square test or Fisher exact test, as appropriate. Continuous variables presented as mean ± SD were compared using Student’s *t* test. Cohen kappa statistics were used to assess inter-observer variability in visual assessment. The area under the receiver operating characteristic curve (AUC) was calculated to determine the best cutoff value for quantitative myocardial CTP parameters for the diagnosis of hemodynamically significant CAD. The diagnostic performances of CTP, CCTA, and CCTA plus CTP were calculated and compared using paired McNemar test. For all diagnostic accuracy parameters, the associated 95% confidence intervals (CIs) were calculated. A *P* < .05 was considered statistically significant.

## 3. Results

### 3.1. Patient characteristics

Table 1 summarizes the patient characteristics. Noted that 58.5% of patients underwent CCTA because of chest pain, and 41.5% of asymptomatic patients underwent CCTA because of known or suspected CAD. No statistical differences were detected between patients with hemodynamically significant coronary artery stenosis and those without hemodynamically significant coronary artery stenosis based on FFR in terms of baseline characteristics. No cardiac events or procedure-related complications were documented during CTP or invasive coronary angiography with FFR. Among the 42 patients, 4 (9.5%) had nonsignificant stenosis (<50%), 28 (66.7%) had single-vessel disease, 9 (21.4%) had 2-vessel disease, and one (2.4%) had 3-vessel disease as detected by invasive coronary angiography. Among 125 coronary vessels, 23 (18.4%) displayed moderate stenosis and 23 (18.4%) exhibited severe stenosis. Twenty (16.0%) of the 125 assessable coronary vessels in 16 patients (38.1%) were categorized as hemodynamically significant based on FFR < 0.8.

**Table 1:** Patient characteristics.

Characteristics	Total patients (N = 42)	Patients with functionally significant coronary artery stenosis (n = 16)	Patients without functionally significant coronary artery stenosis (n = 26)	*P* value
Age (yr)	59.7 ± 8.8	58.0 ± 8.4	60.8 ± 9.1	.324
Male patients	31 (73.8)	12 (75.0)	19 (73.1)	1.000*
Height (cm)	166.0 ± 8.1	165.6 ± 8.6	166.2 ± 7.9	.803
Weight (kg)	69.7 ± 11.4	70.1 ± 11.8	69.5 ± 11.3	.864
BMI (kg/m^2^)	25.2 ± 3.0	25.5 ± 3.0	25.1 ± 3.1	.716
Diabetes	12 (28.6)	4 (25.0)	8 (30.8)	.740*
Hypertension	25 (59.5)	10 (62.5)	15 (57.7)	.758
Stroke	3 (7.1)	2 (12.5)	1 (3.8)	.547*
Current smoking	15 (35.7)	6 (37.5)	9 (34.6)	.850
Symptoms				.892*
No chest pain	17(41.5)	8 (50.0)	9 (36.0)	
Typical chest pain	8 (19.5)	3 (18.8)	5 (20.0)	
Atypical chest pain	13 (31.7)	4 (25.0)	9 (36.0)	
Nonanginal chest pain	3 (7.3)	1 (6.3)	2 (8.0)	
Pretest CAD probability				.843*
Low (<10%)	18 (42.9)	8 (50.0)	10 (38.5)	
Intermediate (10%–90%)	16 (38.1)	5 (31.3)	11 (42.3)	
High (>90%)	8 (19.0)	3 (18.8)	5 (19.2)	
Mean HR during CCTA scan (beats/min)	72.3 ± 14.1	72.8 ± 13.8	72.0 ± 14.5	.870
Mean HR during CTP scan (beats/min)	63.5 ± 10.6	64.2 ± 9.9	63.0 ± 11.1	.739
Coronary artery calcium score	282.4 ± 432.7	357.8 ± 598.1	228.6 ± 264.5	.444

Values are expressed as mean ± standard deviation or n (%).

BMI = body mass index, CAD = coronary artery disease, CCTA = coronary computed tomography angiography, CTP = computed tomography perfusion, HR = heart rate.

* Fisher exact test.

### 3.2. CTA analysis

The interobserver agreement was substantial for image quality (kappa value, 0.675; *P* < .001) and almost perfect for coronary stenosis evaluation (kappa value, 0.940; *P* < .001). The mean image quality score of CCTA was 1.7 ± 0.7 (range, 1–3). There were no cases with nonevaluable image quality. Table S2, Supplemental Digital Content 1, http://links.lww.com/MD/H249 lists the characteristics of CCTA. Eighteen patients (42.9%) had single-vessel disease, 8 (19.0%) had the 2-vessel disease, and 3 (7.1%) had 3-vessel disease on CCTA. Among 125 coronary vessels, 25 (20.0%) had moderate stenosis and 15 (12.0%) had severe stenosis.

### 3.3. CTP analysis

The inter-observer agreement was substantial (kappa value, 0.620; *P* < .001) or almost perfect (kappa value, 0.806; *P* < .001) on the image quality score using static or dynamic CTP. The mean image quality was 1.3 ± 0.6 (range, 1–3) for static CTP and 1.3 ± 0.6 (range, 1–3) for dynamic CTP. The inter-observer agreement was moderate (kappa value, 0.512; *P* < .001) or substantial (kappa value, 0.678; *P* < .001) for determining the presence of perfusion defects using static or dynamic CTP.

**Figure 3.**
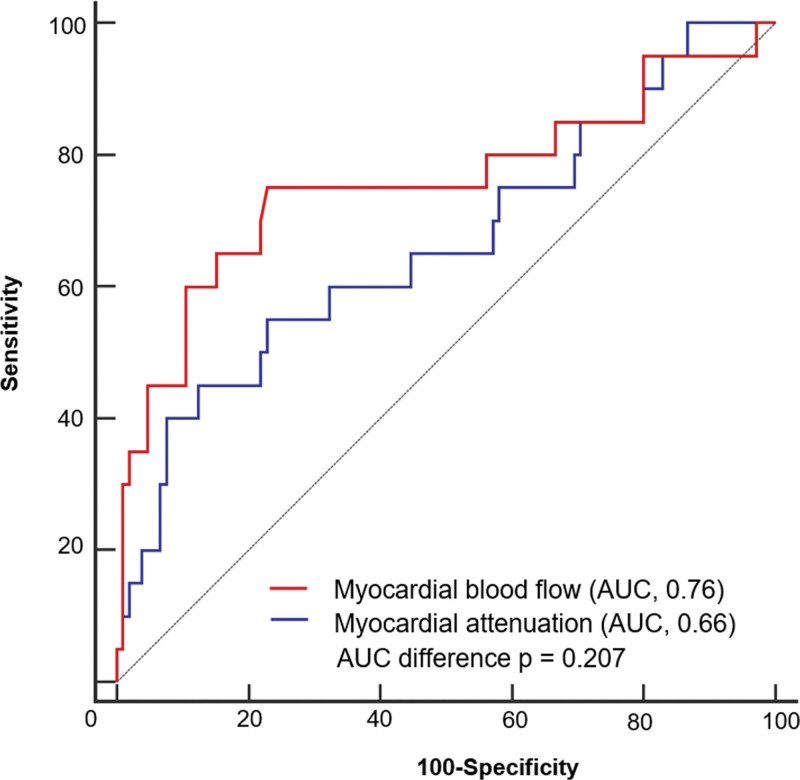
Receiver operating characteristic (ROC) curve and corresponding area under the curve (AUC) describing the diagnostic performance of the quantitative parameters including the myocardial attenuation and myocardial blood flow (MBF) to identify functionally significant coronary artery stenosis at a vessel level. AUCs were 0.66 (95% CI, 0.57–0.75, *P* = .028) for myocardial attenuation and 0.76 (95% CI, 0.67–0.83, *P* < .001) for MBF. AUCs were not significantly different (*P* = .207).

The mean myocardial CT attenuation of ischemic myocardium (86.8 ± 23.9 HU) on static CTP was significantly lower than that of nonischemic myocardium (102.3 ± 20.1 HU, *P* = .003). The MBF of the ischemic myocardium was also significantly lower than that of the nonischemic myocardium (90.4 ± 39.1 mL/100 mL/min vs 123.3 ± 30.9 mL/100 mL/min; *P* < .001). Figure 3 shows the ROC curves for these quantitative parameters, including myocardial CT attenuation and MBF. The calculated AUCs, 0.66 (95% CI, 0.57–0.75; *P* = .028) for myocardial attenuation and 0.76 (95% CI, 0.67–0.83; *P* < .001) for MBF, were not significantly different (*P* = .207).

Table 2 demonstrates the diagnostic performance data of dynamic and static CTPs. The per-vessel sensitivity, specificity, positive predictive value, negative predictive value, and diagnostic accuracy of visual assessment were 40.0%, 98.1%, 80.0%, 89.6%, and 88.8%, respectively, when using static CTP and 50.0%, 98.1%, 83.3%, 91.2%, and 90.4%, respectively, for dynamic CTP. Per-vessel visual analysis of dynamic CTP yielded similar sensitivity (*P* = .530), specificity (*P* = 1.0), and diagnostic accuracy (*P* = .558) to those of static CTP. The per-vessel sensitivity, specificity, positive predictive value, negative predictive value, and diagnostic accuracy of myocardial attenuation using cut-off values of ≤82 HU were 45.0%, 87.6%, 40.9%, 89.3%, and 80.8%, respectively, while those of MBF using cut-off values of ≤101 mL/100 mL/min were 75.0%, 78.1%, 39.5%, 94.3%, and 77.6%, respectively. There was no significant difference in diagnostic performance between static and dynamic CTPs when using visual analysis or quantitative analysis (*P* value range, 0.133–0.765). On combined visual and quantitative analysis, no significant difference in sensitivity (55.0% vs 80.0%, *P* = .096), specificity (87.6% vs 78.1%, *P* = .069), or diagnostic accuracy (82.4% vs 78.4%, *P* = .426) were observed between static and dynamic CTPs. The AUC of visual analysis (0.690 vs 0.740, *P* = .546), quantitative analysis (0.663 vs 0.765, *P* = .203), and combined analysis (0.713 vs 0.790, *P* = .312) were also not significantly different between static and dynamic CTPs for per-vessel analysis.

**Table 2:** Comparison of diagnostic performance among static CTP and dynamic CTP on per-vessel analysis.

Parameters	TP	TN	FP	FN	Sensitivity (%)	Specificity (%)	PPV (%)	NPV (%)	Diagnostic accuracy (%)	AUC
Static CTP										
Visual analysis	8	103	2	12	40.0 (19.1–63.9)	98.1 (93.3–99.8)	80.0 (47.8–94.6)	89.6 (85.7–92.5)	88.8 (81.9–93.7)	0.690 (0.602–0.770)
Quantitative analysis (myocardial attenuation ≤82 HU)	9	92	13	11	45.0 (23.1–68.5)	87.6 (79.8–93.2)	40.9 (25.5–58.3)	89.3 (84.8–92.6)	80.8 (72.8–87.3)	0.663 (0.573–0.745)
Combined visual and quantitative analysis	11	92	13	9	55.0 (32.5–76.9)	87.6 (79.8–93.2)	45.8 (30.7–61.7)	91.1 (86.2–94.3)	82.4 (74.6–88.6)	0.713 (0.625–0.790)
Dynamic CTP										
Visual analysis	10	103	2	10	50.0 (27.2–72.8)	98.1 (93.3–99.8)	83.3 (54.2–95.5)	91.2 (86.9–94.1)	90.4 (83.8–94.9)	0.740 (0.654–0.815)
Quantitative analysis (MBF ≤ 101 mL/100 mL/min)	15	82	23	5	75.0 (50.9–91.3)	78.1 (69.0–85.6)	39.5 (29.6–50.3)	94.3 (88.4–97.2)	77.6 (69.3–84.6)	0.765 (0.681–0.837)
Combined visual and quantitative analysis	16	82	23	4	80.0 (56.3–94.3)	78.1 (69.0–85.6)	41.0 (31.3–51.5)	95.3 (89.5–98.0)	78.4 (70.2–85.3)	0.790 (0.709–0.858)
Static CTP vs Dynamic CTP										
*P* value (visual analysis)					.530	1.000	.845	.683	.558	.546
*P* value (quantitative analysis)					.056	.069	.994	.217	.534	.203
*P* value (combined visual and quantitative analysis)					.096	.069	.786	.259	.426	.312

AUC = area under the curve, CTP = computed tomography perfusion, FN = false negative, FP = false positive, MBF = myocardial blood flow, NPV = negative predictive value, PPV = positive predictive value, TN = true negative, TP = true positive.

**Figure 4.**
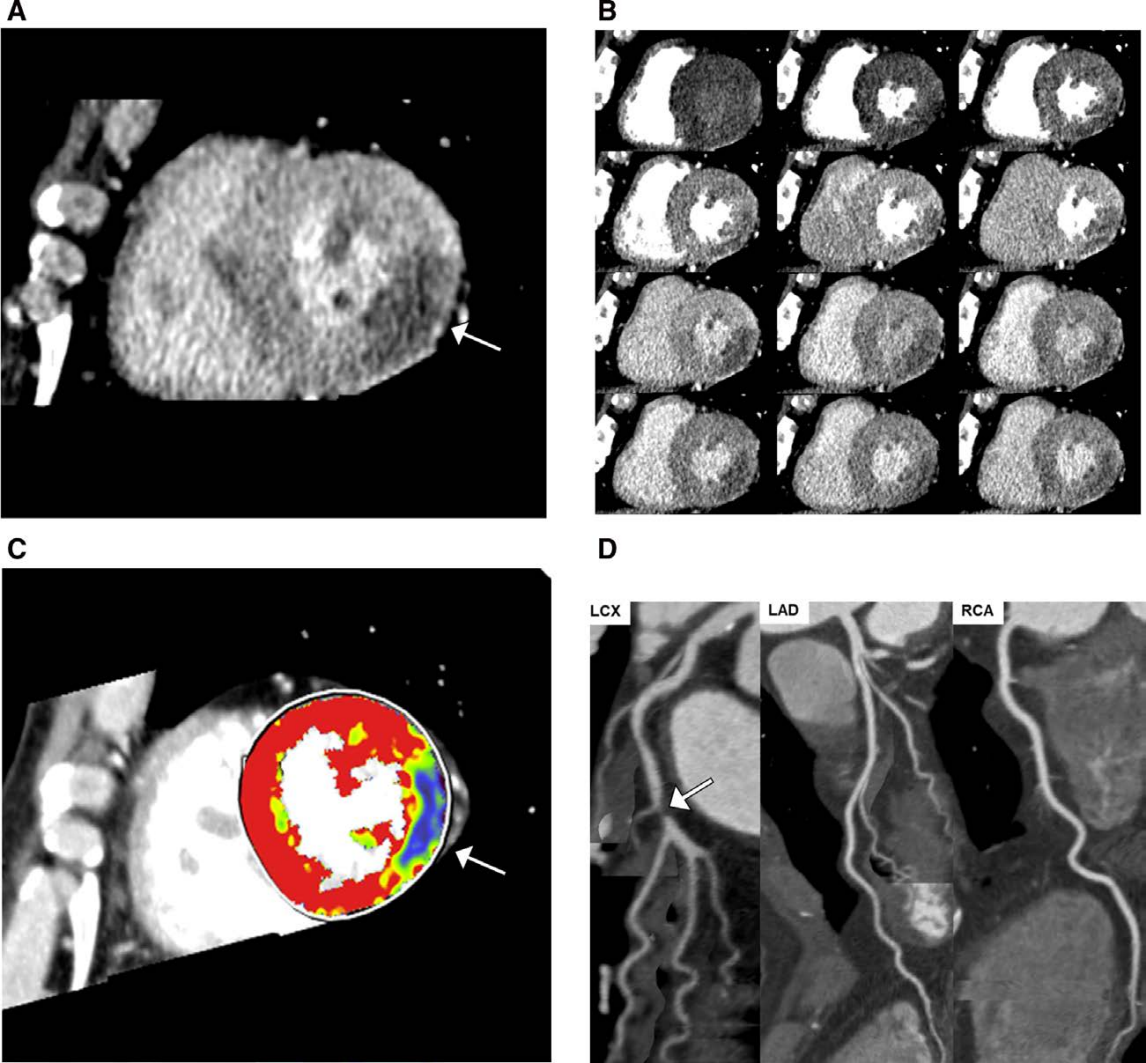
Stress CT myocardial perfusion images in a 53-year-old male patient with distal left circumflex artery stenosis. Virtual static CTP image (A) and dynamic CTP images (B and C) show myocardial perfusion defects in the anterior lateral wall of the middle left ventricle. Virtual static CTP image (A) demonstrates a perfusion defect (arrow) measuring 58.2 HU. Color map (C) shows the reduced myocardial blood flow (31.1 mL/100 mL/min, arrow) in the left circumflex artery territory. Coronary CT angiography (D) demonstrates severe coronary artery stenosis (arrow) in the left circumflex artery. FFR was not measured in the distal left circumflex artery because the degree of stenosis was >90%. CTP = computed tomography perfusion, FFR = fractional flow reserve, HU = hounsfield unit.

Figure 4 demonstrates an example of a diagnosis of hemodynamically significant stenosis using stress CTP.

### 3.4. Additional diagnostic value of CTP over CCTA

Table 3 shows comparison of diagnostic accuracy between CCTA alone (stenosis ≥50%) and combined CCTA with CTP. In a vessel-based analysis, using combined CCTA with static CTP (77.1%–100.0%, all *P* < .001) and combined CCTA with dynamic CTP (77.1% to 94.3%–99.0%, all *P* < .001), significant improvements in specificity were observed. Diagnostic accuracy was also improved in case of combined CCTA with static CTP (77.6% to 88.8%–90.4%; *P*-value range, .003–.018) or dynamic CTP (77.6% to 88.8%–89.6%; *P* value range, .011–.018) over CCTA alone. However, the diagnostic accuracy of combined CCTA and static CTP was not significantly different from that of combined CCTA and dynamic CTP (*P* value range, .679–.844). The sensitivity of CCTA plus MBF (66.0% vs 80.0%, *P* = .325) or CCTA plus combined analysis using dynamic CTP (65.0% vs 80.0%, *P* = .294) was not significantly different compared with that of CCTA alone. However, sensitivity of static CTP (from 80.0% to 30–40%, *P* value range, .002–.024) or visual analysis using dynamic CTP (from 80% to 40%, *P* = .011) combined with CCTA significantly decreased compared to that of CCTA alone. There was no significant difference in diagnostic performance between CCTA plus static CTP and CCTA plus dynamic CTP on per-vessel analysis (*P*-value range, .679–.839).

**Table 3:** Comparison of per-vessel diagnostic performance among CCTA alone, CCTA plus static CTP, and CCTA plus dynamic CTP.

Parameters	TP	TN	FP	FN	Sensitivity (%)	Specificity (%)	PPV (%)	NPV (%)	Diagnostic accuracy (%)	AUC
CCTA (stenosis ≥50%)	16	81	24	4	80.0 (56.3–94.3)	77.1 (67.9–84.8)	40.0 (30.6–50.2)	95.3 (89.3–98.0)	77.6 (69.3–84.6)	0.786 (0.703–0.854)
CCTA + Static CTP										
CCTA + Visual analysis	6	105	0	14	30.0* (11.9–54.3)	100.0* (96.5–100.0)	100.0* (100.0–100.0)	88.2 (84.9–90.9)	88.8* (81.9–93.7)	0.650 (0.560–0.733)
CCTA + Quantitative analysis (Myocardial attenuation ≤82 HU)	8	105	0	12	40.0* (19.1–63.9)	100.0*,† (96.5–100.0)	100.0* (100.0–100.0)	89.7 (86.0–92.6)	90.4* (83.8–94.9)	0.700 (0.612–0.779)
CCTA + Combined visual and quantitative analysis	9	105	0	11	45.0* (23.1–68.5)	100.0*,† (96.5–100.0)	100.0* (100.0–100.0)	90.5 (86.5–93.4)	91.2* (84.8–95.5)	0.725 (0.638–0.801)
CCTA + Dynamic CTP										
CCTA + Visual analysis	8	104	1	12	40.0* (19.1–63.9)	99.0* (94.8–100)	88.9* (51.4–98.4)	89.7 (85.8–92.5)	89.6* (82.9–94.3)	0.695 (0.607–0.774)
CCTA + Quantitative analysis (MBF ≤ 101 mL/100 mL/min)	12	99	6	8	66.0 (36.1–80.9)	94.3*,† (88.0–97.9)	66.7 (46.0–82.5)	92.5 (87.8–95.5)	88.8* (81.9–93.7)	0.771 (0.688–0.842)
CCTA + Visual and quantitative analysis	13	99	6	7	65.0 (40.8–84.6)	94.3*,† (88.0–97.9)	68.4* (48.3–88.6)	93.4 (88.6–96.3)	89.6* (82.9–94.3)	0.796 (0.715–0.863)
CCTA alone vs CCTA + Static CTP										
*P* value (visual analysis)					.002	<.001	.007	.079	.018	.128
*P* value (quantitative analysis)					.011	<.001	.002	.147	.006	.322
*P* value (combined visual and quantitative analysis)					.024	<.001	.001	.202	.003	.475
CCTA alone vs CCTA + Dynamic CTP										
*P* value (visual analysis)					.011	<.001	.009	.148	.011	.27
*P* value (quantitative analysis)					.325	<.001	.062	.428	.018	.826
*P* value (combined visual and quantitative analysis)					.294	<.001	.043	.576	.011	.861
Static CTP vs Dynamic CTP										
*P* value (visual analysis)					.513	.305	.415	.733	.839	.467
*P* value (quantitative analysis)					.104	.013	.068	.465	.679	.437
*P* value (combined visual and quantitative analysis)					.209	.013	.062	.430	.668	.428

AUC = area under the curve, CCTA = coronary computed tomography angiography, CTP = computed tomography perfusion, FN = false negative, FP = false positive, MBF = myocardial blood flow, NPV = negative predictive value, PPV = positive predictive value, TAC = time-attenuation curve, TN = true negative, TP = true positive.

* Significantly different compared to CCTA alone.

† Significantly different between static and dynamic CTPs.

### 3.5. Radiation dose

The mean DLP associated with CCTA and CTP was 234 ± 155.6 mGy and 687 ± 123.6 mGy, respectively. The effective radiation dose was 3.3 ± 2.2 mSv and 9.6 ± 1.7 mSv for CCTA and CTP, respectively.

## 4. Discussion

The present study compared the diagnostic performance of static and dynamic CTPs. The main finding of this study is that combined visual and quantitative analyses were not significantly different between dynamic and static CTP. CTP had additional diagnostic value over stenosis ≥50% using CCTA alone with increased specificity and diagnostic accuracy in patients on per-vessel analysis.

To the best of our knowledge, this is the first quantitative and qualitative CT study that directly compares the diagnostic performance between static and dynamic CTPs. Our study revealed that static and dynamic CTPs have comparable diagnostic accuracy. In particular, the diagnostic accuracy of myocardial attenuation showed no significant difference from that of MBF. This result is similar to a previous report which demonstrated that there was no significant difference in diagnostic accuracy compared with that of a semi-quantitative evaluation and the analysis of one data set acquired at a single time point during peak enhancement of the myocardium.^[13]^

A recent meta-analysis^[21]^ suggested that quantitative dynamic CTP assessment has a higher sensitivity than static CTP. The authors explained that this might be related to easier identification of small perfusion defects from the quantitative assessment through dynamic CTP that cannot be appreciated by visual assessment of static CTP. The tendency of the higher sensitivity of MBF was shown compared to that of visual assessment using static CTP (75.0% vs 45.0%) in this study. However, diagnostic performance was not significantly different between static and dynamic CTPs when comparing the visual, quantitative, and combined visual and quantitative analyses, respectively. To confirm these results, further studies with a larger sample size are needed.

Our results showed that the diagnostic performance of CCTA plus CTP assessment compared with FFR was better than that of CCTA alone. While ≥50% stenosis on CCTA is very sensitive in finding functionally significant coronary stenosis defined as FFR <0.8, it lacks specificity. Although severe coronary calcification is known to affect the diagnostic performance of CCTA with a specificity of approximately 90% in patients with calcium scores of 0 to 499 and 50.6% in patients with calcium scores of 401 to 1000,^[21]^ CTP is not influenced by coronary calcification. Therefore, CCTA complemented by CTP can result in increased specificity. Similar to our study, a previous prospective study evaluated the diagnostic performance of CCTA vs CCTA plus CTP.^[22]^ Rossi et al^[23]^ reported an additional value beyond CCTA in intermediate coronary lesions and revealed that the specificity of CCTA (69%) could be improved by the subsequent use of the MBF index (89%). However, additional radiation and contrast medium exposure of CTP remains issue compared to magnetic resonance imaging, echocardiography, and stress testing without imaging.^[24]^

Our study has a few limitations. First, the myocardial CTP method consisted of only a stress-perfusion protocol. Therefore, the flow reserve could not be calculated and compared. Second, the small sample size might not be sufficient to assess the diagnostic performance. Therefore, future studies with larger sample sizes would be required to validate our results. Finally, the sensitivity of the CTP was relatively low.

In conclusion, static CTP had a diagnostic performance similar to dynamic CTP for the detection of hemodynamically significant coronary stenosis. Stress CTP provided additional diagnostic value over CCTA by increasing the specificity and diagnostic accuracy.

## Acknowledgments

We acknowledge the assistance of Kwang Min Lee (KM consulting) and Pusan National Hospital clinical trial center biostatistics office with the statistical analysis.

## Author contributions

**Conceptualization:** Ji Won Lee, Yeon Hyeon Choe.

**Data curation:** Ji Won Lee, Yeon Hyeon Choe, Ki Seok Choo.

**Formal analysis:** Ji Won Lee, Sung Mok Kim, Jin-Ho Choi, Ki Seok Choo, Chong Eun Lee.

**Funding acquisition:** Yeon Hyeon Choe.

**Investigation:** Ji Won Lee, Sung Mok Kim, Jin-Ho Choi, Seongyong Pak, Ki Seok Choo, Jeong Su Kim, Chong Eun Lee, Yun-Hyeon Kim.

**Methodology:** Ji Won Lee, Sung Mok Kim, Ki Seok Choo, Yun-Hyeon Kim.

**Project administration:** Yeon Hyeon Choe.

**Resources:** Seongyong Pak.

**Software:** Seongyong Pak.

**Supervision:** Yeon Hyeon Choe, Ki Seok Choo, Yun-Hyeon Kim.

**Validation:** Ji Won Lee, Jin-Ho Choi, Jeong Su Kim, Yun-Hyeon Kim.

**Visualization:** Ji Won Lee.

**Writing – original draft:** Ji Won Lee, Ki Seok Choo.

**Writing – review & editing:** Ji Won Lee, Yeon Hyeon Choe, Sung Mok Kim, Jin-Ho Choi, Seongyong Pak, Ki Seok Choo, Jeong Su Kim, Chong Eun Lee, Yun-Hyeon Kim.

## Supplementary Material


