# Supplementary material for: Comparison of diagnostic performance between dynamic versus static adenosine-stress myocardial CT perfusion to detect hemodynamically significant coronary artery stenosis: A prospective multicenter study
Source: Medicine (Baltimore). 2022 Sep 9;101(36):e30477. doi: 10.1097/MD.0000000000030477 (PMC10980466; doi:10.1097/MD.0000000000030477)
Supplement: Supplementary file 1 [file medi-101-e30477-s001.pdf]

Supplementary Table

Supplementary Table 1. Maximum attenuation difference between normal and ischemic myocardium in nine patents

Patient	Age	Sex	Number of datasets	MAD (HU)	Best dataset showing MAD (seconds)
1	53	M	12	61.4	7th (17.5s)
2	49	M	12	51.4	7th (16.4s)
3	61	M	11	60.3	7th (19.9s)
4	65	M	11	31.1	6th (16.1s)
5	71	F	10	25.7	7th (20.9s)
6	56	M	12	48.2	7th (18.5s)
7	58	F	14	34.2	8th (16.3s)
8	54	M	12	38.2	9th (21.0s)
9	73	M	11	49.1	8th (20.4s)

MAD, Maximum attenuation difference between normal and ischemic myocardium
Maximum attenuation difference was shown mostly in 7th datasets (16.1s-21.20s).
